# Interrater reliability of photographic assessment of thyroid eye disease using the VISA classification

**DOI:** 10.1007/s10792-024-02934-z

**Published:** 2024-02-20

**Authors:** Antony C. Boynes, Nicholas J. Enright, Thomas G. Hardy, Jwu Jin Khong

**Affiliations:** 1 Orbital Plastics and Lacrimal Unit, Royal Victorian Eye and Ear Hospital, 32, Gisborne Street, East Melbourne, VIC 3002 Australia; 2 Department of Surgery, University of Melbourne, Centre for Eye Research Australia Ltd, East Melbourne, VIC Australia; 3 Department of Ophthalmology, Austin Health, Heidelberg, VIC Australia; 4 Department of Surgery, University of Melbourne, Royal Melbourne Hospital, Parkville, VIC Australia

**Keywords:** Thyroid eye disease, Machine learning, Interrater reliability, Orbit, Photographic assessment

## Abstract

**Purpose:**

To determine the interrater reliability (IRR) of thyroid eye disease (TED) photographic assessment using the VISA classification. To assess whether a VISA grading atlas improves ophthalmology trainees’ performance in photographic assessment of TED.

**Methods:**

A prospective, partially randomized, international study conducted from September 2021 to May 2022. Online study invitation was emailed to a volunteer sample group of 68 ophthalmology college accredited consultants and trainees, and 6 were excluded from the study. Participants were asked to score 10 patient photographs of TED using only the inflammation and motility restriction components of the VISA classification. IRR was compared between groups of practitioners by their level of experience. A clinical activity grading atlas was randomized to 50% of the ophthalmology trainees.

**Results:**

Overall rater ICC was 0.96 for inflammation and 0.99 for motility restriction. No statistically significant difference in IRR between rater groups was identified. Trainees with a grading atlas had the highest IRR for inflammation (ICC = 0.95). Each subcomponent of the inflammation and motility restriction components of VISA classification had an ICC considered good to excellent. The mean overall rater score was 4.6/9 for inflammation and 3.5/12 for motility restriction. For motility restriction there was a reduced mean score variance among all raters when scoring photographs with more severe motility restriction.

**Conclusion:**

IRR using the inflammation and motility restriction components of the VISA classification was excellent. A VISA grading atlas improved trainee performance in grading inflammation.

**Supplementary Information:**

The online version contains supplementary material available at 10.1007/s10792-024-02934-z.

## Introduction

Thyroid eye disease (TED) is an autoimmune disease often causing permanent facial disfigurement and substantially affecting patients’ quality of life and daily function [1–3]. While intravenous glucocorticoids remains a first line medical treatment for TED in the active phase in many parts of the world, about 35% of cases do not respond, and 11% showed inflammation reactivation [4–7]. Increasingly immunotherapies are utilized in the treatment of TED to not only suppress inflammation, but to modify disease severity with the aim of reversing proptosis and improving ocular motility [8–11]. One of the first monoclonal antibodies, teprotumumab, has been FDA-approved for the treatment of TED based on interventional trials [8, 9, 12]. One key aspect of conducting treatment trials in TED and to monitor treatment response in clinical care, is to ensure the parameters used to define treatment response are reliable and reproducible [13].

One of the challenges of TED progression and treatment response assessment is how to classify and grade its various clinical manifestations [14, 15]. Currently periocular inflammation is standardly assessed by the Clinical Activity Score (CAS) or a derivative of it [16–21]. Ocular motility assessment is less standardised where grading a range of eye duction varied from subjective grading from 0 to 4, where 0 is zero duction and 4 is full duction or 0 to − 4, where 0 is full duction and − 4 is no duction, by corneal reflex position to estimate the degree of eye duction, to more vigorous and laborious methods using kinetic perimetry [22–26]. Interobserver reliability and therefore validation of all of these methods remain to be explored.

The vision, inflammation, strabismus, appearance/exposure (VISA) classification categorizes four specific end points of TED and summarizes these in a clinical form useful for grading specific measurements and for guiding management [17, 27, 28]. The advantage of the VISA classification is that it grades both disease severity and activity using subjective and objective inputs. The clinician decides on disease progression if there is interval change in individual sections for example, a change of 2 or more on the inflammatory score or 15° change in motility restriction (Table 1) [17, 29]. This allows the VISA classification to acknowledge different aspects of eye and periocular changes that can be disproportionately affected by TED. For example, a patient may present with predominantly extraocular muscle involvement or predominantly fat hypertrophy which may not be reflected in grading systems such as NOSPECS which assume a linear progression of disease [15]. The VISA classification was initially validated in a pilot study using ten patients referred with TED who were assessed by two clinicians. This study found that the scores correlated 100% for vision and appearance, 80% for inflammation, and 90% for strabismus [27]. Mawn et al. 2018 conducted a study to validate the reliability of three different grading scales used to measure soft tissue changes in TED (VISA, NOSPECS, CAS). They found that intrarater reliability was better than interrater reliability (IRR) for all scales and that the VISA classification met threshold for agreement for conjunctival and eyelid erythema but not for caruncular oedema [30].

**Table 1:** VISA classification scoring for inflammation and motility restriction

VISA iInflammation	Scoring
Caruncular oedema	0: absent, 1: present
Chemosis	0: absent, 1: conjunctiva lies behind the grey line of the lid, 2: conjunctiva lies anterior to the grey line of the lid
Conjunctival redness	0: absent, 1: present
Lid redness	0: absent, 1: present
Lid oedema—upper, lower	0: absent, 1: present without festoon, 2: present with festoon
Total score	0–10

VISA motility restriction	Scoring
Upgaze, downgaze, abduction, adduction	0: > 45°, 1: 30–45°, 2: 15–30°, 3: 0–15°
Total score	0–3

Studies have shown significant interrater and intrarater variability in ability to assess TED patients with in-person assessment using the VISA classification [30]. These studies recommended assessing expert agreement using photographic evaluation of patients with TED [30]. Photographic assessment is important as it enables re-assessment of specific variables at different time intervals, greater participant recruitment, and development of machine-learning based image interpretation in future studies.

The purpose of this study is to determine the accuracy of photographic assessment of TED using the VISA classification [17]. We compare the IRR of assessment between orbital and oculoplastic subspecialists, subspecialists in other areas, general ophthalmologists, and ophthalmology trainees. In addition, we assess whether a TED grading atlas improves trainee performance in assessing photographs of TED.

## Methods

### Study design and ethical approval

A prospective, partially randomized study between September 2021 and May 2022 participants were asked to score 10 patient photographs of TED using only the inflammation and motility restriction components of the VISA classification. The Royal Victorian Eye and Ear Hospital’s Human Research Ethics Committee granted ethics approval (approval number 21/1515HL) before the initiation of the study. This study adhered to the tenets of the Declaration of Helsinki. Participants were asked to respond to 13 questions including grading 10 photographs from 7 patients with TED and 3 questions on participant demographics/clinical experience. Photographs were provided in isolation and there was no additional patient information included. The 10 TED photographs were selected by one of the senior authors who subspecializes in TED and included a range of severity for 5 inflammation and 5 motility restriction photographs and written patient consent was provided. Photographs were viewed in a randomized sequence for each rater to minimize observer fatigue and learning. Raters were asked to grade the severity of inflammation and motility restriction using the VISA classification. An abridged version of The Graves Orbitopathy Clinical Evaluation Atlas by EUGOGO [23] was randomly provided to 50% of the trainees via the survey software program to provide instructions on grading TED activity using the inflammation and motility restriction components; none of the other rater groups were provided with an atlas. Our abridged version contains the identical inflammation photographs with adapted instructions to score using VISA grading as well as additional photographs on scoring motility restriction.

For the purposes of this study inflammation was scored as follows—caruncular oedema 0–1, chemosis 0–2, conjunctival redness 0–1, lid redness 0–1, upper lid oedema 0–2, and lower lid oedema 0–2, addition of each of these components giving maximum total score of 9. Motility restriction was scored as per the VISA classification system: 0 =  > 45degrees of duction, 1 = 30–45 degrees, 2 = 15–30 degrees, 3 =  < 15 degrees; upgaze 0–3, downgaze 0–3, abduction 0–3, and adduction 0–3, addition of each of these components giving a maximum total score of 12.

### Participants

The online study invitation was emailed to ophthalmology college accredited consultants and trainees. The study invitation was distributed internationally via the Royal Australian and New Zealand College of Ophthalmologists, Australian and New Zealand Society of Ophthalmic Plastic Surgeons, The Royal College of Ophthalmologists—England. Locally the study invitation was distributed to practitioners at The Royal Victorian Eye and Ear Hospital, Melbourne, Australia, and Sydney Eye Hospital, Sydney, Australia. Incomplete responses were excluded. Raters were grouped by subspecialty into the following rater groups: orbital and oculoplastic subspecialists, other subspecialty, general ophthalmologists, and ophthalmology trainees. ‘Other subspecialist’ was defined as an ophthalmologist who had completed fellowship into a subspecialty other than orbital and oculoplastics.

### Data collection and analysis

Data was exported from the SurveyMonkey online platform to a Microsoft Excel spreadsheet (Version 16.66.1). Two-way mixed effect absolute agreement model intraclass correlation coefficients were calculated using IBM SPSS Statistics (Version 28). IBM SPSS Statistics (Version 28) was used to calculate a one-way ANOVA test to calculate inflammation and motility restriction mean severity scores and variance for all raters and between group and within group sum of squares and *F* values were calculated for each TED photograph. A result was considered statistically significant where the source of variation indicated a between group sum of squares greater than the within group sum of squares and this corresponded to an *F*-value greater than *F*-critical value and a *p*-value less than 0.05. A two-tailed independent-samples *T*-test was used to assess the effectiveness of the TED grading atlas among the trainee cohort. Consensus for interpretation of ICC is as follows < 0.5 poor, 0.5–0.75 moderate, 0.75–0.9 good, and > 0.9 excellent [31–35]. A statistician was consulted about the appropriate methods for analysis.

## Results

### Rater demographics and clinical experience

From a total of 68 responses, 6 were incomplete and excluded from the study. The remaining 62 respondents included 18 orbital and oculoplastic subspecialists (29.0%), 9 other subspecialists (14.5%), 14 general ophthalmologists (22.5%) and 21 ophthalmology trainees, randomized to 10 with (16.1%) and 11 without atlas (17.7%).

Across all rater groups, most participants managed less than 5 TED cases per week. Orbital and oculoplastic subspecialist raters on average treated the most TED cases of any group including 27.7% seeing 5–10 TED cases per week and 11.1% seeing 11–15 cases per week. Participants from the General Ophthalmologist group had the greatest number of total clinic years’ experience, with 28.5% having 16–20 years and 64.2% having greater than 20 years of clinical experience. Table 2 shows the demographics and clinical experience of each of these groups.

**Table 2:** Demographic characteristics of participants

	Orbital and oculoplastics (*n* = 18)	Other subspecialist (*n* = 9)	General Ophthalmologist (*n* = 14)	Trainee-no atlas (*n* = 11)	Trainee-with atlas (*n* = 10)
*Clinical experience (years)*					
< 5	0 (0%)	0 (0%)	1 (7.1%)	9 (81.8%)	8 (80.0%)
5–10	7 (38.8%)	1 (11.1%)	0 (0%)	2 (18.1%)	2 (20.0%)
11–15	6 (33.3%)	2 (22.2%)	0 (0%)	0 (0%)	0 (0%)
16–20	2 (11.1%)	0 (0%)	4 (28.5%)	0 (0%)	0 (0%)
> 20	3 (16.6%)	6 (66.6%)	9 (64.2%)	0 (0%)	0 (0%)
*TED cases seen per week*					
< 5	11 (61.1%)	8 (88.8%)	14 (100%)	9 (81.8%)	10 (100%)
5–10	5 (27.7%)	1 (11.1%)	0 (0%)	2 (18.1%)	0 (0%)
11–15	2 (11.1%)	0 (0%)	0 (0%)	0 (0%)	0 (0%)

### Severity of inflammation and motility restriction

For all raters, mean severity score for inflammation for all patient photographs was 4.6/9, ranging from 3.0 to 5.7 for each patient photographs (Table S1). For all raters, the mean severity of motility restriction score for all patient photographs was 3.6/12, ranging from 1.2 to 6.4 for each patient photographs (Table S2). When comparing all raters, variance between mean severity scores for inflammation ranged from 1.5 to 3.4 and motility variance for mean motility restriction ranged from 0.7 to 1.5. The source of this variance reflects differences between individual rater scores. For motility restriction there was a reduced variance among all raters when scoring more severe motility restriction (*p*-value < 0.05). There was no reduction in variance between raters when scoring inflammation regardless of severity.

### Interrater reliability for inflammation and motility restriction

The ICCs for inflammation and motility restriction are shown in Table 3. Intraclass correlation coefficients for all groups were considered good to excellent; the ICC for inflammation assessment was 0.96, and for motility restriction was 0.99 for all raters. There was no statistically significant difference in between rater groups for scoring of inflammation or motility restriction using the VISA classification. For all rater groups the ICCs for inflammation photographs 1, 4, 6 and 7 were excellent (ICC > 0.9) and uniform across rater groups for inflammation scoring, with minor variance noted in photograph 3 across rater groups. For motility restriction assessment, the ICCs for photographs 2, 8 and 9 were excellent (ICC > 0.9) and uniform across all rater groups, with moderate to good agreement for photographs 5 and 10. (Refer to supplementary Tables S3 and S4) Intraclass correlation coefficients for each subcomponent of inflammation (caruncular oedema, chemosis, conjunctival redness, lid redness, upper lid oedema, lower lid oedema) and motility restriction scoring (upgaze, downgaze, abduction, adduction) were good to excellent for all raters (Table 4). Gaze positions had the highest ICC (ICC 0.97–0.99), followed by conjunctival redness, chemosis and upper eyelid oedema; the lowest ICC was for eyelid erythema at 0.82. Orbital and oculoplastic surgeons had the highest ICC with near perfect agreement for inflammation in all photographs. Four of the 5 motility restriction photographs had excellent IRR. Photograph 10 had moderate to good IRR for motility restriction.

**Table 3:** Intraclass correlation coefficients (ICC) for each of the rater groups for inflammation and motility restriction

Rater group	VISA component	
	Inflammation (ICC)	Motility restriction (ICC)
All raters (*n* = 62)	0.96	0.99
Orbital and oculoplastics (*n* = 18)	0.89	0.99
Subspecialist other (*n* = 9)	0.87	0.96
General ophthalmologist (*n* = 14)	0.86	0.98
Trainee – no atlas (*n* = 11)	0.87	0.94
Trainee – with atlas (*n* = 10)	0.95	0.97

**Table 4:** Intraclass correlation coefficients (ICC) for each subsection of the inflammation and motility restriction VISA components for all raters

VISA component	ICC
Caruncular oedema	0.93
Chemosis	0.97
Conjunctival redness	0.97
Lid redness	0.82
Upper lid oedema	0.96
Lower lid oedema	0.94
Upgaze	0.99
Downgaze	0.99
Abduction	0.97
Adduction	0.98

### Effect of a grading atlas on interrater reliability

**Fig. 1:**
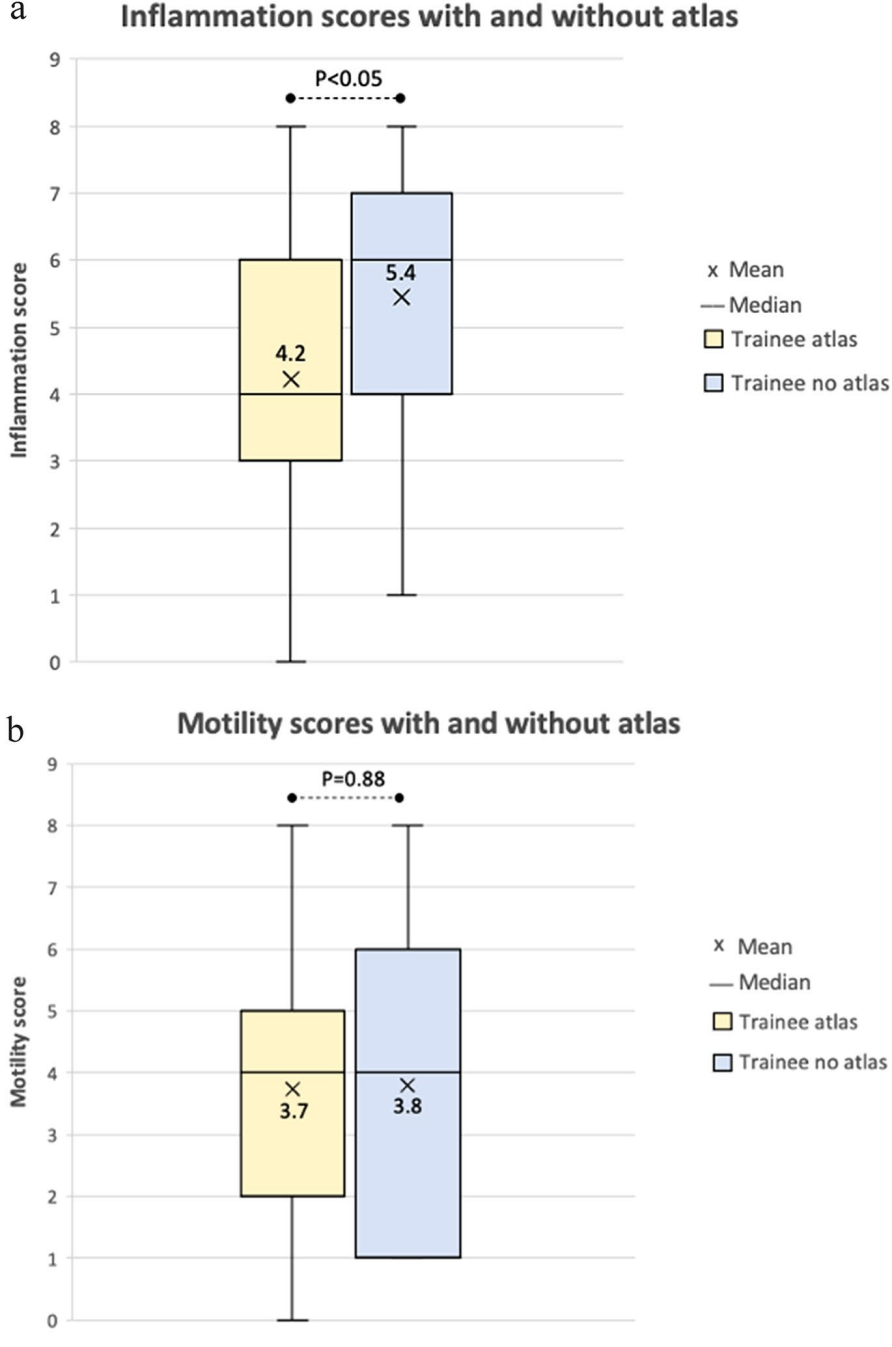
**a** VISA inflammation scores for all patient photographs for trainees with a grading atlas (yellow) and trainees without the use of a grading atlas (blue). **b** VISA motility restriction scores for all patient photographs for trainees with a grading atlas (yellow) and trainees without the use of a grading atlas (blue)

There was a statistically significant difference in the mean severity score of inflammation for the trainee with atlas group (mean = 4.2) and the trainee group without the atlas (mean = 5.4, *p*-value < 0.05), Fig. 1a. The trainee with atlas group scored inflammation closer to the overall rater mean of 4.6. This suggests trainees without the use of a grading atlas tended to overestimate inflammation. The trainee with atlas group had greater IRR when assessing mild motility restriction. For example, the ICC motility restriction scores for photograph 5 and 10 for the with atlas group were 0.97 and 0.79 respectively, in comparison to 0.83 and 0.68 for the without atlas group. There was no statistically significant difference in mean severity scores for motility restriction for the trainee with atlas group (mean = 3.7) and trainee without atlas group (mean = 3.8), Fig. 1b. One-way ANOVA testing showed no significant differences in variance between the trainee with or without atlas groups for inflammation or motility restriction scoring.

## Discussion

This study found that photographic assessment’s IRR for inflammation was excellent for raters of all skill levels. When comparing IRR between the different rater groups the trainee cohort with the atlas had the highest ICC for inflammation at 0.95 and general ophthalmologists performed the least well (ICC = 0.86). For the inflammation section of the VISA classification most scoring components had excellent IRR (ICC > 0.9) except perhaps eyelid redness. Nevertheless, lid redness still had good reliability with an ICC of 0.82. VISA scoring of lid redness is binary, either 0 (no erythema) or 1 (definite erythema). Redness must exceed generalized facial redness to score; however, this is highly subjective. The findings of this study are consistent with a previous study which found excellent IRR when using digital images to measure eyelid fissure height in patients with Graves’ Ophthalmopathy [36]. However, IRR with photographic assessment may not be directly comparable with in person assessment. Mawn et al. showed a binary (present/absent) scoring system for the inflammation components of VISA had moderate agreement (mean fraction of agreement = 0.74) [30, 37, 38]. This difference in findings is likely due to different statistical measurements as well as the study design comparing two raters at different institutions whereas our study assessed the IRR of all 62 raters. Subject variance may have also contributed to the difference in findings, our study had a range of severity which would lead to a greater ICC (Tables S1 and S2). Although, it may also reflect differences in reliability between photographic assessment and in person assessment. Studies on the validity of photographic assessment including machine learning assisted systems of ophthalmic conditions have suggested while there were some useful correlations, the 2 methods are not currently interchangeable [39–43]. A study comparing digital image measurement with clinical measurement of eyelid fissure height in Graves’ Ophthalmopathy showed fair to moderate agreement when assessing eyelid position in terms of palpebral fissure and marginal light reflex distance. A possible explanation for this discrepancy is the static eyelid position in the photographic measurement compared to the dynamic position with in person assessment [36].

There was no significant difference between the ICCs of the orbital and oculoplastics group where raters had 11–15 years of experience and had the highest clinical case load of TED patients, when compared to the trainee without atlas group where 80% of raters had < 5 years’ experience. The trainee with atlas group had the highest ICC for inflammation (ICC = 0.95). This supports the use of a grading atlas to assist consistency of scoring between raters. Our study revealed the IRR for all raters was excellent for motility restriction (ICC = 0.99). There was no statistically significant difference between each rater group. In agreement, each component of upgaze, downgaze, abduction and adduction also had excellent IRR of above 0.95 for all raters. Our findings are consistent with a previous study that found absolute percentage agreement of 90% for motility restriction [27]. However, comparison to these findings is limited due to the previous study utilizing in person assessment and only comparing two raters. Our current study showed a positive correlation between motility restriction severity and ICC, where ICC increases as motility restriction increases. This suggests that the level of interrater agreement increases with increasing motility restriction. Motility restriction photographs 5 and 10 had the lowest mean score (1.7/12 and 1.2/12, respectively); this corresponded to the lowest ICC scores for all raters for these photographs. These findings are consistent with a previous study on reliability of measuring ductions using the light reflex method which found an interrater overall coefficient of repeatability (CR) of 9.6 degrees with 95% confidence [22]. Therefore, smaller changes in duction measurements < 9.6 degrees in less severe motility restriction will likely have lower IRR. There is no consensus on what an acceptable value of CR for a measurement tool and it is up to the discretion of the clinician. Intraclass correlation coefficients and CR are not directly comparable, and both may be utilized.

This study showed improved IRR for scoring inflammation and motility restriction with the use of a grading atlas in the trainee cohort. The trainee with atlas group had a greater ICC for both inflammation and motility restriction when compared to the trainee without atlas group. In comparison, the trainee without atlas group tended to overestimate inflammation; for example, periorbital swelling from orbital fat hypertrophy may be confused with eyelid oedema [44–46]. Dickinson and Perros developed a grading atlas for TED due to difficulties with assessment and meaningful interpretation of research [23, 47]. The atlas illustrates targeted qualitative assessment for soft tissue grading of TED. Validation of this tool shows that soft tissue grading can be performed more reliably with the use of a comparative photographic atlas [23]. Our modified grading atlas has clear definitions with diagrammatic representation on VISA scoring and was designed to be used as a reference at the time of VISA scoring. Having standardized explanations of scoring at the time of assessment likely reduces variability. The grading atlas appears to enhance accuracy in assessing inflammation, and especially useful in grading less severe motility restriction. To improve IRR, we recommend institutional consensus on a standardized TED assessment tool amongst practitioners. In institutions that treat large numbers of TED patients a VISA grading atlas may be beneficial to less experienced staff.

There are some limitations to this study. The ICC is strongly influenced by the variance amongst the population in which it is assessed and is calculated as a ratio: (variance of interest)/(variance of interest + unwanted variance) [34, 48, 49]. Therefore ICCs measured amongst different study populations might not be directly comparable. When ICCs for rater groups are calculated on an individual photograph the variance of interest may be much smaller than the variance from all photographs collectively. This may explain the decreased ICC for individual photographs for each of the rater groups [34, 50, 51]. Our study contained photographs of variable size and resolution which may have led to increase variance between scores. Attempts were made to select good quality photographs of differing disease severity based on expert opinion. To better compare the accuracy of photographic assessment of TED a study should compare in person clinical examination with photographic assessment. When collecting data on clinical experience we did not request raters to specify their familiarity with the VISA classification. As this was an international study some raters may be more familiar with other assessment tools. This may have led to raters’ level of clinical experience being incorrectly classified. The VISA grading atlas was only randomized to 50% of the trainee group. To better assess the utility of a grading atlas a future study may have the same group of raters assess the same patient photograph with and without the atlas to assess intraobserver reliability. This study only assessed the inflammation and motility restrictions of the VISA classification.

## Conclusion

To our knowledge this is the first study assessing the accuracy of photographic assessment of TED. It is also the first study to directly compare raters of different levels of clinical experience and the effect of a grading atlas on IRR. In conclusion, we found the inflammation and ocular motility grading of the VISA classification to be reliable amongst raters of all levels of clinical experience. It showed that photographic assessment of inflammation and motility restriction in TED had excellent IRR. We would recommend a future study to compare the accuracy of photographic assessment with live clinical assessment. This will be an important comparison with future advances in machine learning-systems development for the detection of TED.

## Supplementary Information

Below is the link to the electronic supplementary material.

Supplementary file1 (TIFF 483 KB)

Supplementary file2 (TIFF 696 KB)

Supplementary file3 (DOCX 17 KB)

## References

[1] Patel A, Yang H, Douglas RS (2019) A new era in the treatment of thyroid eye disease. Am J Ophthalmol 208:281–288 31377284 10.1016/j.ajo.2019.07.021

[2] Wang Y, Padnick-Silver L, Francis-Sedlak M, Holt RJ, Foley C, Douglas RS (2022) Inflammatory and noninflammatory thyroid eye disease: comparison of disease signs, symptoms, and quality of life in patients in the United States. Endocr Pract 28(9):842–846 35714862 10.1016/j.eprac.2022.06.003

[3] Otto EA, Ochs K, Hansen C, Wall JR, Kahaly GJ (1996) Orbital tissue-derived T lymphocytes from patients with Graves’ ophthalmopathy recognize autologous orbital antigens. J Clin Endocrinol Metab 81(8):3045–3050 8768872 10.1210/jcem.81.8.8768872

[4] Mourits MP, Koornneef L, Wiersinga WM, Prummel MF, Berghout A, van der Gaag R (1989) Clinical criteria for the assessment of disease activity in Graves’ ophthalmopathy: a novel approach. Br J Ophthalmol 73(8):639–644 2765444 10.1136/bjo.73.8.639 PMC1041835

[5] Gontarz-Nowak K, Szychlińska M, Matuszewski W, Stefanowicz-Rutkowska M, Bandurska-Stankiewicz E (2020) Current knowledge on graves' orbitopathy. J Clin Med. 10(1). 10.3390/jcm10010016 PMC7793490 33374706

[6] Bahn RS (2010) Graves’ ophthalmopathy. N Engl J Med 362(8):726–738 20181974 10.1056/NEJMra0905750 PMC3902010

[7] Tsui S, Naik V, Hoa N, Hwang CJ, Afifiyan NF, Sinha Hikim A et al (2008) Evidence for an association between thyroid-stimulating hormone and insulin-like growth factor 1 receptors: a tale of two antigens implicated in Graves’ disease. J Immunol 181(6):4397–4405 18768899 10.4049/jimmunol.181.6.4397 PMC2775538

[8] Douglas RS, Kahaly GJ, Patel A, Sile S, Thompson EHZ, Perdok R et al (2020) Teprotumumab for the treatment of active thyroid eye disease. N Engl J Med 382(4):341–352 31971679 10.1056/NEJMoa1910434

[9] Ugradar S, Kang J, Kossler AL, Zimmerman E, Braun J, Harrison AR et al (2022) Teprotumumab for the treatment of chronic thyroid eye disease. Eye (Lond) 36(8):1553–1559 34244669 10.1038/s41433-021-01593-z PMC9307784

[10] Denisova K, Barmettler A (2021) Evaluating the thyroid eye disease patient. Int Ophthalmol Clin 61(2):33–52 33743527 10.1097/IIO.0000000000000351

[11] Barbesino G, Salvi M, Freitag SK (2022) Future projections in thyroid eye disease. J Clin Endocrinol Metab 107(Suppl_1):S47–S56. 36346684 10.1210/clinem/dgac252 PMC9359449

[12] Smith TJ, Kahaly GJ, Ezra DG, Fleming JC, Dailey RA, Tang RA et al (2017) Teprotumumab for thyroid-associated ophthalmopathy. N Engl J Med 376(18):1748–1761 28467880 10.1056/NEJMoa1614949 PMC5718164

[13] Ma L, Hui S, Li Y, Hou Z, Liu Z, Chang Q et al (2022) Different characteristics of orbital soft tissue expansion in graves orbitopathy: extraocular muscle expansion is correlated to disease activity while fat tissue volume with duration. J Craniofac Surg 33(8):2354–2359 35882057 10.1097/SCS.0000000000008751

[14] Li Q, Ye H, Ding Y, Chen G, Liu Z, Xu J et al (2017) Clinical characteristics of moderate-to-severe thyroid associated ophthalmopathy in 354 Chinese cases. PLoS ONE 12(5):e0176064 28472149 10.1371/journal.pone.0176064 PMC5417486

[15] Van Dyk HJ (1981) Orbital Graves' disease. A modification of the "NO SPECS" classification. Ophthalmology 88(6):479–483. 6894971

[16] Bartalena L, Kahaly GJ, Baldeschi L, Dayan CM, Eckstein A, Marcocci C et al (2021) The 2021 European Group on Graves’ orbitopathy (EUGOGO) clinical practice guidelines for the medical management of Graves’ orbitopathy. Eur J Endocrinol 185(4):G43–G67 34297684 10.1530/EJE-21-0479

[17] Dolman PJ (2018) Grading severity and activity in thyroid eye disease. Ophthalmic Plast Reconstr Surg 34(4S Suppl 1):S34–S40. 29952931 10.1097/IOP.0000000000001150

[18] Mourits MP, Prummel MF, Wiersinga WM, Koornneef L (1997) Clinical activity score as a guide in the management of patients with Graves’ ophthalmopathy. Clin Endocrinol (Oxf) 47(1):9–14 9302365 10.1046/j.1365-2265.1997.2331047.x

[19] Rundle FF, Wilson CW (1945) Development and course of exophthalmos and ophthalmoplegia in Graves’ disease with special reference to the effect of thyroidectomy. Clin Sci 5(3–4):177–194 21011937

[20] Perros P, Baldeschi L, Boboridis K, Dickinson AJ, Hullo A, Kahaly GJ et al (2006) A questionnaire survey on the management of Graves’ orbitopathy in Europe. Eur J Endocrinol 155(2):207–211 16868132 10.1530/eje.1.02201

[21] Bartalena L, Baldeschi L, Dickinson A, Eckstein A, Kendall-Taylor P, Marcocci C et al (2008) Consensus statement of the European Group on Graves’ orbitopathy (EUGOGO) on management of GO. Eur J Endocrinol 158(3):273–285 18299459 10.1530/EJE-07-0666

[22] Dolman PJ, Cahill K, Czyz CN, Douglas RS, Elner VM, Feldon S et al (2012) Reliability of estimating ductions in thyroid eye disease: an International Thyroid Eye Disease Society multicenter study. Ophthalmology 119(2):382–389 21959369 10.1016/j.ophtha.2011.07.011

[23] Dickinson AJ, Perros P (2001) Controversies in the clinical evaluation of active thyroid-associated orbitopathy: use of a detailed protocol with comparative photographs for objective assessment. Clin Endocrinol (Oxf) 55(3):283–303 11589671 10.1046/j.1365-2265.2001.01349.x

[24] Wiersinga WM, Perros P, Kahaly GJ, Mourits MP, Baldeschi L, Boboridis K et al (2006) Clinical assessment of patients with Graves’ orbitopathy: the European Group on Graves’ Orbitopathy recommendations to generalists, specialists and clinical researchers. Eur J Endocrinol 155(3):387–389 16914591 10.1530/eje.1.02230

[25] Dagi LR, Zoumalan CI, Konrad H, Trokel SL, Kazim M (2011) Correlation between extraocular muscle size and motility restriction in thyroid eye disease. Ophthalmic Plast Reconstr Surg 27(2):102–110 21383547 10.1097/IOP.0b013e3181e9a063

[26] Bontzos G, Papadaki E, Mazonakis M, Maris TG, Tsakalis NG, Drakonaki EE et al (2022) Extraocular muscle volumetry for assessment of thyroid eye disease. J Neuroophthalmol 42(1):e274–e280 34629402 10.1097/WNO.0000000000001339

[27] Dolman PJ, Rootman J (2006) VISA classification for graves orbitopathy. Ophthalmic Plast Reconstr Surg 22(5):319–324 16985411 10.1097/01.iop.0000235499.34867.85

[28] Dolman PJ (2012) Evaluating Graves’ orbitopathy. Best practice and research. Clin Endocrinol Metabolism 26:229–248. 10.1016/j.beem.2011.11.007 22632361

[29] Hutchings KR, Fritzhand SJ, Esmaeli B, Koka K, Zhao J, Ahmed S, et al (2023) Graves' Eye disease: clinical and radiological diagnosis. Biomedicines 11(2). 10.3390/biomedicines11020312 PMC9953404 36830848

[30] Mawn LA, Dolman PJ, Kazim M, Strianese D, Genol I, Chong KKL et al (2018) Soft tissue metrics in thyroid eye disease: an international thyroid eye disease society reliability study. Ophthalmic Plast Reconstr Surg 34(6):544–546 29465482 10.1097/IOP.0000000000001080

[31] Koo TK, Li MY (2016) A guideline of selecting and reporting intraclass correlation coefficients for reliability research. J Chiropr Med 15(2):155–163 27330520 10.1016/j.jcm.2016.02.012 PMC4913118

[32] Bartko JJ (1966) The intraclass correlation coefficient as a measure of reliability. Psychol Rep 19(1):3–11 5942109 10.2466/pr0.1966.19.1.3

[33] Schuck P (2004) Assessing reproducibility for interval data in health-related quality of life questionnaires: which coefficient should be used? Qual Life Res 13(3):571–586 15130022 10.1023/B:QURE.0000021318.92272.2a

[34] Liljequist D, Elfving B, Skavberg RK (2019) Intraclass correlation—a discussion and demonstration of basic features. PLoS ONE 14(7):e0219854 31329615 10.1371/journal.pone.0219854 PMC6645485

[35] Li L, Zeng L, Lin ZJ, Cazzell M, Liu H (2015) Tutorial on use of intraclass correlation coefficients for assessing intertest reliability and its application in functional near-infrared spectroscopy-based brain imaging. J Biomed Opt 20(5):50801 25992845 10.1117/1.JBO.20.5.050801

[36] Edwards DT, Bartley GB, Hodge DO, Gorman CA, Bradley EA (2004) Eyelid position measurement in Graves’ ophthalmopathy: reliability of a photographic technique and comparison with a clinical technique. Ophthalmology 111(5):1029–1034 15121384 10.1016/j.ophtha.2003.08.027

[37] Hunt RJ (1986) Percent agreement, Pearson’s correlation, and kappa as measures of inter-examiner reliability. J Dent Res 65(2):128–130 3455967 10.1177/00220345860650020701

[38] McHugh ML (2012) Interrater reliability: the kappa statistic. Biochem Med (Zagreb) 22(3):276–282 23092060 PMC3900052

[39] Karlin J, Gai L, LaPierre N, Danesh K, Farajzadeh J, Palileo B, Taraszka K, Zheng J, Wang W, Eskin E, Rootman D (2022) Ensemble neural network model for detecting thyroid eye disease using external photographs. Br J Ophthalmol. 10.1136/bjo-2022-321833 10.1136/bjo-2022-321833 36126104

[40] Moon JH, Shin K, Lee GM, Park J, Lee MJ, Choung H et al (2022) Machine learning-assisted system using digital facial images to predict the clinical activity score in thyroid-associated orbitopathy. Sci Rep 12(1):22085 36543834 10.1038/s41598-022-25887-8 PMC9772205

[41] Schulz CB, Clarke H, Makuloluwe S, Thomas PB, Kang S (2023) Automated extraction of clinical measures from videos of oculofacial disorders using machine learning: feasibility, validity and reliability. Eye (Lond), pp 1–7. 10.1038/s41433-023-02424-z PMC9891656 36725916

[42] Gulshan V, Peng L, Coram M, Stumpe MC, Wu D, Narayanaswamy A et al (2016) Development and validation of a deep learning algorithm for detection of diabetic retinopathy in retinal fundus photographs. JAMA 316(22):2402–2410 27898976 10.1001/jama.2016.17216

[43] Hood DC, De Moraes CG (2018) Efficacy of a deep learning system for detecting glaucomatous optic neuropathy based on color fundus photographs. Ophthalmology 125(8):1207–1208 30032794 10.1016/j.ophtha.2018.04.020

[44] Wiersinga WM, Bartalena L (2002) Epidemiology and prevention of Graves’ ophthalmopathy. Thyroid 12(10):855–860 12487767 10.1089/105072502761016476

[45] Wilson WB, Prochoda M (1995) Radiotherapy for thyroid orbitopathy. Effects on extraocular muscle balance. Arch Ophthalmol 113(11):1420–1425. 10.1001/archopht.1995.01100110080029 7487604

[46] Mimura M, Yang PT, Ko AC, Korn BS, Kikkawa DO (2020) Analysis of periorbital soft tissue in thyroid eye disease. Ophthalmic Plast Reconstr Surg 36(1):30–33 31567914 10.1097/IOP.0000000000001450

[47] Wiersinga WM, Prummel MF, Mourits MP, Koornneef L, Buller HR (1991) Classification of the eye changes of Graves’ disease. Thyroid 1(4):357–360 1841734 10.1089/thy.1991.1.357

[48] Bobak CA, Barr PJ, O’Malley AJ (2018) Estimation of an inter-rater intra-class correlation coefficient that overcomes common assumption violations in the assessment of health measurement scales. BMC Med Res Methodol 18(1):93 30208858 10.1186/s12874-018-0550-6 PMC6134634

[49] Shrout PE, Fleiss JL (1979) Intraclass correlations: uses in assessing rater reliability. Psychol Bull 86(2):420–428 18839484 10.1037//0033-2909.86.2.420

[50] Qin S, Nelson L, McLeod L, Eremenco S, Coons SJ (2019) Assessing test-retest reliability of patient-reported outcome measures using intraclass correlation coefficients: recommendations for selecting and documenting the analytical formula. Qual Life Res 28(4):1029–1033 30547346 10.1007/s11136-018-2076-0 PMC6439259

[51] Mehta S, Bastero-Caballero RF, Sun Y, Zhu R, Murphy DK, Hardas B et al (2018) Performance of intraclass correlation coefficient (ICC) as a reliability index under various distributions in scale reliability studies. Stat Med 37(18):2734–2752 29707825 10.1002/sim.7679 PMC6174967

