# Supplementary figures and images for: Interrater reliability of photographic assessment of thyroid eye disease using the VISA classification
Source: Int Ophthalmol. 2024 Feb 20;44(1):98. doi: 10.1007/s10792-024-02934-z (PMC10879244; doi:10.1007/s10792-024-02934-z)

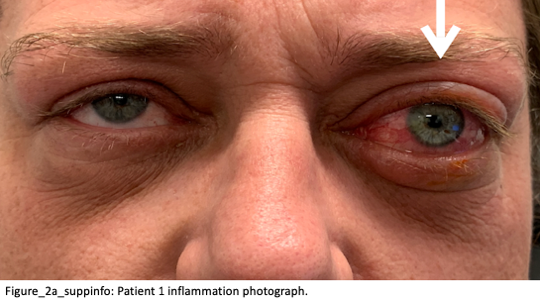

Supplement: Supplementary file 1 — Supplementary file1 (TIFF 483 KB) [file 10792_2024_2934_MOESM1_ESM.tiff]

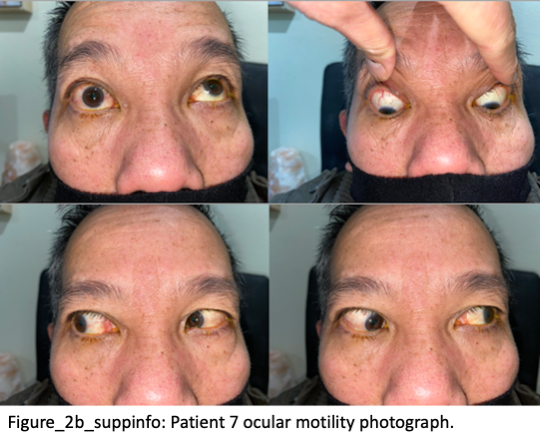

Supplement: Supplementary file 2 — Supplementary file2 (TIFF 696 KB) [file 10792_2024_2934_MOESM2_ESM.tiff]
