# Supplementary material for: Interrater reliability of photographic assessment of thyroid eye disease using the VISA classification
Source: Int Ophthalmol. 2024 Feb 20;44(1):98. doi: 10.1007/s10792-024-02934-z (PMC10879244; doi:10.1007/s10792-024-02934-z)
Supplement: Supplementary file 3 — Supplementary file3 (DOCX 17 KB) [file 10792_2024_2934_MOESM3_ESM.docx]

**Supplemental Material**

| **Patient photograph** | **Count** | **Average** | **Variance** |
| --- | --- | --- | --- |
| Photo 1 | 62 | 5.7 | 1.5 |
| Photo 3 | 62 | 3.0 | 2.9 |
| Photo 4 | 62 | 5.3 | 3.4 |
| Photo 6 | 62 | 4.9 | 2.6 |
| Photo 7 | 62 | 4.2 | 1.9 |

Table S1: One-way ANOVA analysis results for mean inflammation scores for all raters.

Count = the number of raters included; Average = mean inflammation severity score using the VISA classification; Variance = the average of the squared differences from the mean inflammation score.

| **Patient photograph** | **Count** | **Average** | **Variance** |
| --- | --- | --- | --- |
| Photo 2 | 62 | 4.9 | 0.7 |
| Photo 5 | 62 | 1.7 | 1.5 |
| Photo 8 | 62 | 6.4 | 1.4 |
| Photo 9 | 62 | 3.6 | 0.9 |
| Photo 10 | 62 | 1.2 | 2.2 |

Table S2: One-way ANOVA analysis results for mean motility restriction scores for all raters.

Count = the number of raters included; Average = mean inflammation severity score using the VISA classification; Variance = the average of the squared differences from the mean inflammation score.

| **Rater Group** | **Photo 1** | **Photo 3** | **Photo 4** | **Photo 6** | **Photo 7** |
| --- | --- | --- | --- | --- | --- |
| Orbital and oculoplastics (n=18) | 1.0 | 0.97 | 0.99 | 0.99 | 0.99 |
| Subspecialist other (n=9) | 0.99 | 0.94 | 0.96 | 0.98 | 0.99 |
| General ophthalmologist (n=14) | 0.99 | 0.91 | 0.97 | 0.95 | 0.98 |
| Trainee – no atlas (n=11) | 0.99 | 0.94 | 0.98 | 0.99 | 0.97 |
| Trainee – with atlas (n=10) | 0.97 | 0.87 | 0.98 | 0.96 | 0.96 |

Table S3: Intraclass correlation coefficients (ICC) for each of the rater groups for inflammation for each patient photograph.

| **Rater Group** | **Photo 2** | **Photo 5** | **Photo 8** | **Photo 9** | **Photo 10** |
| --- | --- | --- | --- | --- | --- |
| Orbital and oculoplastics (n=18) | 1.0 | 0.97 | 1.0 | 0.99 | 0.85 |
| Subspecialist other (n=9) | 0.98 | 0.89 | 0.96 | 0.99 | 0.78 |
| General ophthalmologist (n=14) | 0.98 | 0.79 | 0.98 | 0.97 | 0.67 |
| Trainee – no atlas (n=11) | 1.0 | 0.83 | 0.99 | 1.0 | 0.68 |
| Trainee – with atlas (n=10) | 0.99 | 0.97 | 0.99 | 0.98 | 0.79 |

Table S4: Intraclass correlation coefficients (ICC) for each of the rater groups for motility restriction for each patient photograph.
